# Interstitial lung disease associated with adjuvant and neoadjuvant chemotherapy in early breast cancer

**DOI:** 10.1186/s12957-021-02289-0

**Published:** 2021-06-11

**Authors:** Kenji Tezuka, Kotaro Miura, Yusuke Nakano, Takahiro Ueda, Kyoko Yagyu, Shimako Matsuyama, Masami Shirai, Hiroshi Okuda, Miho Ujikawa, Takayo Ota

**Affiliations:** 1 Department of Breast Surgery, Izumi City General Hospital, 4-5-1 Wake, Izumi, Osaka, 594-0073 Japan; 2 Department of Surgery, Izumi City General Hospital, 4-5-1 Wake, Izumi, Osaka, 594-0073 Japan; 3 Department of Medical Oncology, Izumi City General Hospital, 4-5-1 Wake, Izumi, Osaka, 594-0073 Japan; 4 Department of Respiratory Medicine, Izumi City General Hospital, 4-5-1 Wake, Izumi, Osaka, 594-0073 Japan; 5 Nursing Department, Izumi City General Hospital Izumi, 4-5-1 Wake, Izumi, Osaka, 594-0073 Japan; 6 Department of Pharmacy, Izumi City General Hospital, 4-5-1 Wake, Izumi, Osaka, 594-0073 Japan

**Keywords:** Breast cancer, Dose-dense chemotherapy, Interstitial lung disease, *Pneumocystis jirovecii* pneumonia

## Abstract

**Background:**

Interstitial lung disease (ILD) is a rare adverse event in patients receiving adjuvant or neoadjuvant chemotherapy (NAC) for breast cancer. Few studies have reported the frequency of ILD in detail, and only small numbers of cases have been described in the literature.

Given these previous findings concerning ILD, we retrospectively examined the clinicopathological characteristics of five cases of ILD who had received epirubicin and cyclophosphamide (EC) and compared their findings with non-ILD cases.

**Methods:**

The present single-center retrospective study included breast cancer patients who underwent adjuvant chemotherapy or NAC at our hospital between January 2014 and January 2021.

**Results:**

Thirty-nine patients who had received EC for operable breast cancer were enrolled in this study. ILD developed 5 out of 39 patients (12.8%). The incidence of ILD in patients with non-dose-dense (dd) or dd chemotherapy was statistically significantly different (*p* = 0.0149). ILD occurred in three patients during dd EC treatment and two during weekly paclitaxel (wPTX) after dd EC. ILD was detected in one patient with high Krebs von den Lungen-6 (KL-6) levels, in two patients with continuous pyrexia, and in two patients from computed tomography imaging, which was taken to estimate the efficacy of chemotherapy, in two patients. Three of the 5 ILD patients underwent bronchoalveolar lavage, and 2 of these patients were diagnosed with *Pneumocystis jirovecii* pneumonia (PCP). There were no cases of serious ILD that required steroid pulse therapy.

**Conclusions:**

Dd chemotherapy may be associated with an increased ILD frequency, which may reflect developing PCP. Careful monitoring and a timely diagnosis are useful for detecting early-stage ILD.

## Background

Interstitial lung disease (ILD) is a large heterogenous group of pulmonary diseases that involve the interstitium of the lungs [1, 2]. Injury to the alveolar epithelial cells initiates an inflammatory response. Inflammatory cells activate fibroblasts in the interstitium, which damage the extracellular matrix permanently and prevent gas exchange in the lungs. The causes of ILD can be classified as idiopathic or non-idiopathic. Drug-induced interstitial lung disease (DILD) is one of the non-idiopathic ILDs.

In breast cancer patients receiving adjuvant or neoadjuvant chemotherapy (NAC), ILD is considered a rare adverse effect with a potentially fatal prognosis. Few studies have reported the frequency of ILD in detail, and only small numbers of cases have been described in the literature. In pivotal phase III clinical trials, with standard adjuvant chemotherapies, such as adriamycin and cyclophosphamide (AC) [3], epirubicin and cyclophosphamide (EC) [4], docetaxel and cyclophosphamide [5], or AC followed by paclitaxel or docetaxel [6], no ILDs were reported as adverse events, with the exception of one case of pneumonia in a patient who received EC [4]. Given these previous findings concerning ILD, we retrospectively examined the clinicopathological characteristics of five cases of ILD who had received EC and compared their findings with non-ILD cases.

## Methods

The present single-center retrospective study included breast cancer patients who underwent adjuvant or NAC at our hospital between January 2014 and January 2021. The ethics committee of our institution approved this study.

The study protocol was approved by the Ethics Committee of Izumi City General Hospital (20-J17, 23 October 2020).

Age, estimated glomerular filtration rate (eGFR), smoking history (renal dysfunction and smoking history are known as a risk factor for ILD [7, 8]), stage, estrogen receptor (ER) status, progesterone receptor (PgR) status, human epidermal growth factor receptor 2 (HER2) overexpression, and menopausal status were studied as patient background and analyzed retrospectively. ER and PgR positive were defined as an expression by immunochemistry (IHC) in more than 10% of the cancer cells. HER2 overexpression was evaluated as positive by IHC with a score of 3+ or 2+ with a positive fluorescence in situ hybridization test.

The adjuvant or neoadjuvant chemotherapy regimens included tri-weekly or dose-dense (dd) chemotherapy, such as EC, or dd EC followed by weekly paclitaxel (wPTX) or triweekly docetaxel. NAC was administered when the clinicopathological risk was estimated high (e.g., tumors ≥ 2 cm, hormone receptor negative, HER2 overexpression were existed). Since April 2018, we implemented the dd EC regimen, and administered especially if nodal status was positive, or hormone negative with high proliferative breast cancer.

Patients received four cycles of EC chemotherapy (E, 90 mg/m^2^; C 600 mg/m^2^). Dd chemotherapy every 2 weeks 4 cycles of EC. All drugs were administered via intravenous infusion on day 1 of each cycle. Prophylactic pegfilgrastim (3.6 mg) was administered via subcutaneous injection on day 3 for all dd regimens. Trastuzumab (HER) alone or in combination with pertuzumab (PER) was administered concurrently during wPTX or triweekly docetaxel in patients with the overexpression of HER2.

The diagnosis of ILD was made by computed tomography (CT). ILD was defined as an interstitial shadow on CT, and was classified into four patterns according to previous reports [9]: (i) diffuse alveolar damage (DAD)-like pattern, (ii) chronic interstitial pneumonia-like pattern, (iii) eosinophilic pneumonia-like pattern, (iv) organizing pneumonia (OP)-like pattern, and (v) hypersensitivity reaction (HR)-like pattern.

*Pneumocystis jirovecii* pneumonia (PCP) was diagnosed with (i) positive polymerase chain reaction from bronchoalveolar lavage (BAL) and (ii) high serum β-D glucan levels.

Adverse events (AEs) were graded according to the common terminology criteria for adverse events 5.0 (CTCAE) [10]. The term “ILD” is considered to be in the same category as pneumonitis in the present report.

Comparisons between two groups were performed using Mann-Whitney test, Pearson’s chi-square test, and Fisher’s exact test. P values of < 0.05 were considered to indicate statistical significance. Data were analyzed with using GraphPad Prism version 8 (La Jolla, CA, USA).

To examine a frequency of ILDs in published literatures, we reviewed literatures and pivotal clinical trials. Using the terms, “adriamycin,” “epirubicin,” “cyclophosphamide.” “dose dense.” “breast cancer,“ “interstitial lung disease,” “pneumonitis.” we searched PubMed up to 21 April 2021 to identify published articles on neoadjuvant/adjuvant chemotherapies associated with ILD in early breast cancer. We included studies written in English. We excluded studies using other treatments, such as using liposomal doxorubicin or included radiotherapy. To review, we included papers reporting phase II or phase III clinical trials.

## Results

### Patient characteristics

Thirty-nine patients who had received EC for operable breast cancer were enrolled in this study. The characteristics of the patients are shown in Table 1. Five out of 39 patients developed ILD (12.8%). The median ages of patients with and without ILD were 55 and 55 (range 38–63 and 29–74) years, respectively. None of the enrolled patients with ILD had a history of smoking. There were no marked differences in the eGFR value, hormonal, HER2, or menopausal statuses of the groups, whereas the incidence of ILD in patients with non-dd or dd chemotherapy was statistically significantly different (*p* = 0.0149). Five of the 18 (27.8%) patients who received dd EC developed ILD.

**Table 1:** Patient characteristics with and without ILD (*n* = 39)

	With ILD (*n* = 5)	Without ILD (*n* = 34)	p value^b)^
**Age (years)**			
Median (range)	55 (38–63)	55 (29–74)	NS
**eGFR (mL/min)**			
Median (range)	90 (60–95)	79.5 (48–120)	NS
**Smoking history**			
Yes	0	4	
No	5	30	NS
**Stage**			
I	0	7	
IIA	2	13	
IIB	2	4	
IIIA	1	6	
IIIB	0	1	
IIIC	0	3	NS
**Estrogen receptor**^a)^			
Positive	4	20	
Negative	1	14	NS
**Progesterone receptor**^a)^			
Positive	3	12	
Negative	2	22	NS
**HER 2 status**			
Positive	0	15	
Negative	5	19	NS
**Menopausal status**			
Premenopausal	1	13	
Postmenopausal	4	21	NS
**dd regimen administered**			
Yes	5	13	
No	0	21	< .05

*ILD* interstitial lung disease, *eGFR* estimated glomerular filtration rate, *HER2* human epidermal growth factor receptor 2, *dd* dose-dense chemotherapy, *NS* not significant

^a^Defined as an expression by immunochemistry in more than 10% of the cancer cells

^b^< 0.05 were considered to indicate statistical significance

### Incidence and characteristics of ILD

Regimens stratified by the presence of ILD are shown in Table 2. ILD occurred in three patients during dd EC and two during wPTX after dd EC.

**Table 2:** Administered regimens with and without ILD (*n* = 39)

Administered regimen	With ILD	Without ILD
EC only	0	1
EC followed by wPTX	0	9
dd EC only	3	0
dd EC followed by wPTX	2	9
EC followed by wPTX with HER	0	6
EC followed by triweekly docetaxel with HER	0	1
EC followed by wPTX with HER and PER	0	4
ddEC followed by wPTX with HER	0	1
ddEC followed by wPTX with HER and PER	0	3

*ILD* interstitial lung disease, *E* epirubicin, *C* cyclophosphamide, *wPTX* weekly paclitaxel, *dd* dose dense, *HER* trastuzumab, *PER* pertuzumab

The clinical characteristics and outcomes of the five patients with ILD are shown in Table 3. ILD was detected in one patient with high Krebs von den Lungen-6 (KL-6) levels, two with continuous pyrexia, and two by performing CT to estimate the anti-cancer effect after EC. Case numbers 3 and 4 in Table 3 were accompanied by a low level of percutaneous oxygen saturation. All patients were considered to have grade 1 or 2 ILD. One patient recovered with drug withdrawal alone. No patients required the administration of intravenous steroid pulse therapy.

**Table 3:** Clinical characteristics and outcomes of 5 patients with ILD (*n* = 5)

Case	Diagnostic opportunity	SpO2^a)^ (%)	KL-6^a)^ (U/mL)	Grade^b)^	CT findings	BAL and its diagnosis	Therapy for ILD
1	Continuous pyrexia	97	316	2	HR	Done, PCP	PSL + TMP/SMX
2	Continuous pyrexia	98	468	1	HR	Done, Negative	None
3	High KL-6, low SpO2	94	716	2	HR	None	PSL
4	E-CT^c)^, low SpO2	94	338	2	OP	None	PSL
5	E-CT	98	346	2	HR	Done, PCP	TMP/SMX

*ILD* interstitial lung disease, *SpO2* percutaneous oxygen saturation, *KL-6* serum Krebs von den Lungen-6, *CT* computed tomography, *BAL* bronchoalveolar lavage sampling, *HR* hypersensitivity reaction-like pattern, *PCP Pneumocystis jiroveci* pneumonia, *PSL* orally prednisolone (a day), *TMP/SMX* orally trimethoprim-sulfamethoxazole (a day), *OP* organizing pneumonia-like pattern

^a^Values were at the onset of ILD. Standard value; SpO2 ≥ 95%, KL-6 < 500 U/mL

^b^ILD (pneumonitis) grade as in common terminology criteria for adverse events 5.0

^c^CT to estimate the anti-cancer effect after epirubicin and cyclophosphamide

**Fig. 1:**
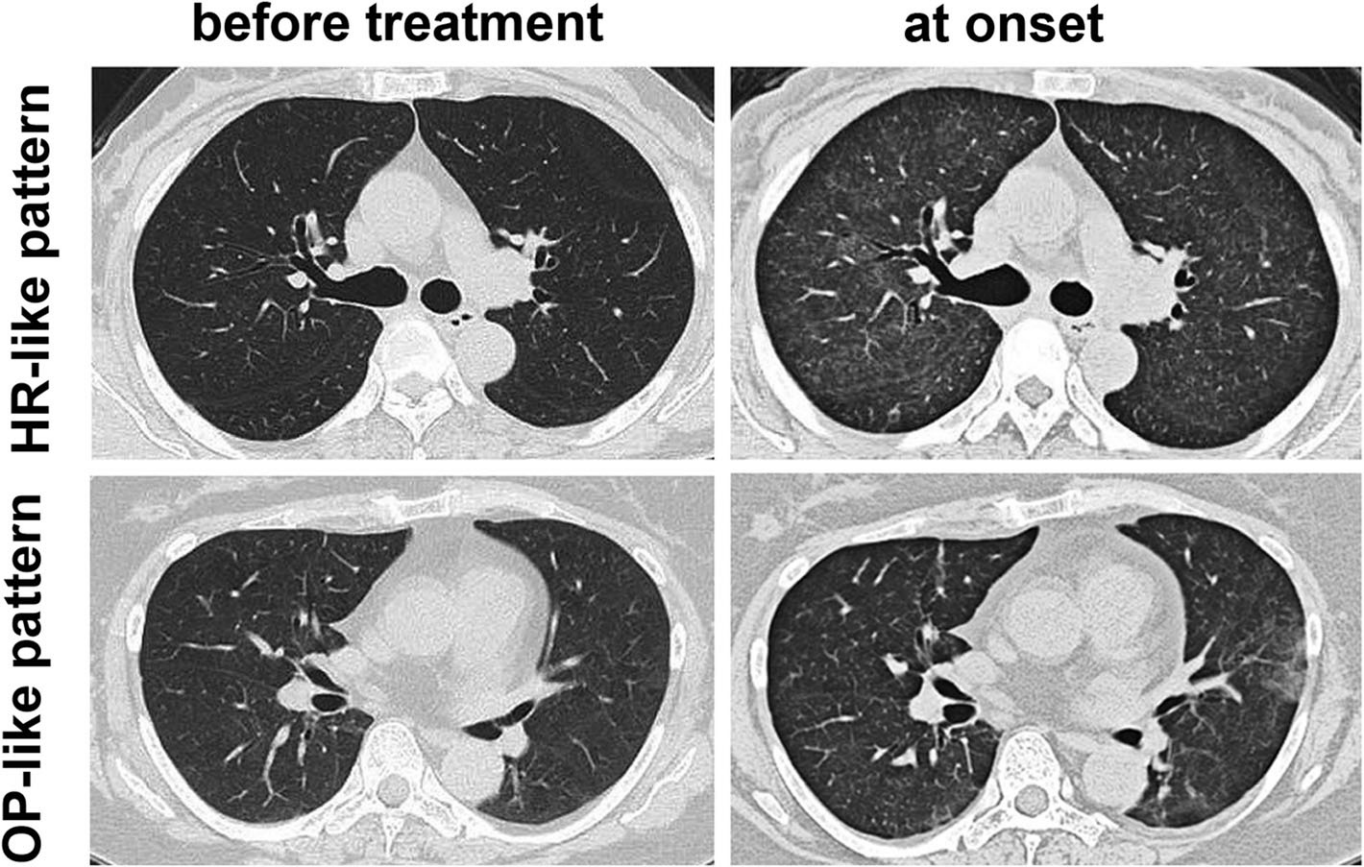
Computed tomography of the chest before neoadjuvant chemotherapy and at the onset of interstitial lung disease in a 63-year-old female (with a hypersensitivity reaction-like pattern similar to that seen in case number 3) and in a 57-year-old female (with an organizing pneumonia-like pattern similar to that seen in case number 4)

Four of the five patients showed an HR-like CT pattern. Representative CT images are shown in Fig. 1. No patients showed any obvious findings on chest X-ray, whereas CT showed bilateral diffuse ground-glass opacity.

Three of the 5 ILD patients underwent BAL, 2 of these patients were diagnosed with PCP and were orally treated with trimethoprim-sulfamethoxazole (720 mg and 3600 mg, respectively, daily). BAL culturing revealed that the remaining one was negative for *P. jiroveci* and *Mycoplasma*. *Candida* antigen was not measured in any cases because they were judged to have mild ILD based on CT.

Regarding the subsequent clinical course, case 3 discontinued preoperative chemotherapy, and then operations were performed after they had recovered from ILD. Cases 1, 4, and 5 resumed wPTX postoperatively after preoperative EC and surgery. Case 2 developed ILD during preoperative wPTX and also recovered with drug withdrawal alone and resumed the administration of the remaining paclitaxel prior to surgery.

**Fig. 2:**
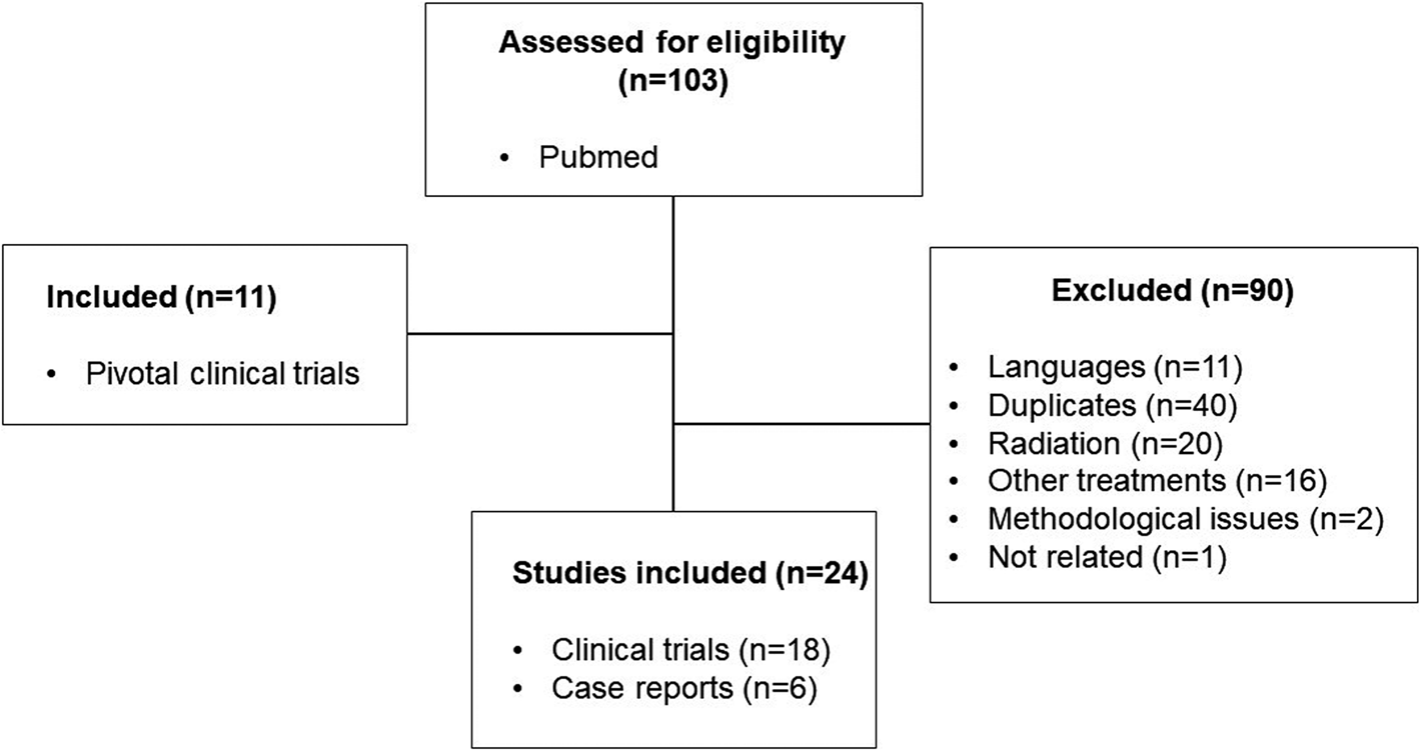
Consort diagram for the literature search

Following the strategies to examine ILD cases in the literature, we retrieved 18 clinical trials and 6 case reports from PubMed (Fig. 2). The result of reviewing literatures is summarized as Table 4 [3–6, 11–30].

**Table 4:** ILD associated with neo/adjuvant AC or (F)EC chemotherapy in early breast cancer

Chemotherapy	Author	Year	ILD	Cause
**Clinical trials**				
AC	Fisher B et al. [3]	1990	NA	
EC	Fisher B et al. [4]	2001	NA	
AC→T, ddAC→T	Citron ML et al. [11]	2003	NA	
ddFEC	Dang CT et al. [12]	2004	9%	
AC→T vs →TH	Romond EH et al. [13]	2005	0% vs 0.4%, 0.6%	
FEC	Venturini M et al. [14]	2005	0%	
ddFEC	Venturini M et al. [14]	2005	< 1%	
AC	Jones SE et al. [5]	2006	NA	
AC→T	Sparano JA et al. [6]	2008	< 0.5%	
ddEC→T	Dang C et al. [15]	2008	0%	
EC→T vs EC→TH	Shimizu et al. [16]	2010	NA	
T→FEC	Gonzalez-Angulo AM et al. [17]	2014	0%	
AC→T vs →TH	Waks AG et al. [18]	2015	0% vs 0%	
ddAC→T vs →TH	Waks AG et al. [18]	2015	0% vs 0.6%	PCP
dd(F)EC→T, (F)EC→T	Del Mastro L et al. [19]	2015	NA	
ddEC→D, FEC	Foukakis T et al. [20]	2016	NA	
(F)AC, (F)EC→TH, DH vs (F)AC, (F)EC→THP or DHP	von Minckwitz G et al. [21]	2017	NA	
AC, EC→DC'H vs AC, EC→DC'HP	von Minckwitz G et al. [21]	2017	NA	
ddAC, ddEC→T	Takabatake D et al. [22]	2018	NA	
ddEC→Next treatment	Morita S et al. [23]	2018	3.9%	PCP
nab-PTX→FEC	Kin T et al. [24]	2020	NA	
**Case reports**				
ddAC	Tolaney SM et al. [25]	2006	1 case	PCP
dd (detailed regimen, not mentioned)	Tolaney SM et al. [25]	2006	1 case	PCP
FEC→T	Kawajiri H et al. [26]	2013	5 cases	
FEC	Shinohara A et al. [27]	2013	1 case	PCP
ddAC→T	Bielopolsky D et al. [28]	2017	3 cases	
EC→H	Sugaya A et al. [29]	2017	1 case	
ddAC	Khoo C et al. [30]	2019	1 case	PCP

*Abbreviations*: *A* doxorubicin, *E* epirubicin, *C* cyclophosphamide, *C'* carboplatin, *D* docetaxel, *dd* dose-dense, *F* fluorouracil, *H* trastuzumab, *ILD* interstitial lung disease, *NA* not assessed, *PCP* pneumocystis jirovecii pneumonia, *T* paclitaxel, *TH* paclitaxel and trastuzumab, *THP* paclitaxel, trastuzumab, and pertuzumab

In phase II or phase III clinical trials, 55.6 % of publications (10 out of 18) did not assess ILDs. In ILD-reported clinical trials, all except two publications reported less than 1% of incidence. As case reports, 13 cases were identified in our literature search.

## Discussion

Dd chemotherapy has been popular since it was introduced in the early 2000s and is now a standard treatment for patients with high-risk breast cancer, as several randomized controlled studies have revealed that its survival benefits are superior to those of standard chemotherapy [11, 20, 22]. In three phase III trials, in adjuvant settings, ILDs were not reported in either the dd or control groups [11, 14, 19]. In these trials, doxorubicin, cyclophosphamide followed by docetaxel [11], or fluorouracil, epirubicin, and cyclophosphamide (FEC) [14], or FEC or EC followed by paclitaxel [19] were administered for chemotherapy. In contrast, in a phase II trial of dd FEC followed by weekly alternate taxane treatment (paclitaxel or docetaxel) as adjuvant therapy, 4 of 44 patients (9%) developed ILD (pneumonitis) during dd FEC [12], and in a Brazilian population, the frequency of ILD in patients who received wPTX (80 mg/m^2^) was as high as 4.2% [31].

In the case of HER2-positive breast cancer, no cases of ILD were reported in a phase II trial of EC followed by PTX and HER [16]. In the B-31 trial, which investigated AC followed by PTX and HER, 4 of 864 patients developed ILD, one of whom died. In the N9381 trial, 5 of 814 patients in the trastuzumab group developed grade 3 ILD or pulmonary infiltration, one of whom died [13]. In patients who received HER and PER in addition to several adjuvant regimens, only grade 3 adverse events were reported; none of these patients developed ILD [21]. The incidence of ILDs in this study was approximately 13% and tended to be higher in patients who received a dd regimen. Although this incidence was relatively high in comparison to previously reported studies, all patients developed grade 1 or 2 ILD and recovered without intravenous steroid pulse therapy.

It is difficult to determine the culprit of ILDs in patients who receive multiple drugs simultaneously. Any drugs are capable of inducing ILDs; however, reports of FEC- or EC-related ILDs are very rare in the relevant literature. Paclitaxel-induced ILDs are also rare, with a reported incidence of 0.7–12% [28, 32]. In the present study, ILDs were identified during dd EC as well as during PTX following dd EC. Both agents might be responsible for DILDs.

Dd chemotherapy increases the incidence of PCP, one of representative causes of ILD. PCP has been reported in several patients receiving dd chemotherapy for early breast cancer [30, 33]. In a large cohort study, the overall incidence of PCP among 2057 patients treated with dd AC was 0.6%, whereas no PCP was diagnosed in 1001 patients treated with non-dd AC [18]. The total dose of steroids (as prophylaxis against nausea during the administration of AC) over 8 weeks of dd chemotherapy in comparison to 12 weeks of standard chemotherapy might explain the increased incidence of PCP [18]. In the present study, 5 out of 18 (27.8%) patients developed ILDs with dd chemotherapy. We also identified two cases of PCP in patients who received dd chemotherapy. We used dexamethasone (4 mg, twice daily) on days 2–3, which might have influenced the rate of PCP infection. It is important to obtain BAL samples to distinguish DILD from PCP.

In most cases of DILD, CT findings are characterized by a bilateral diffuse, extensive patchy, reticular, or ground-glass appearance, with a pattern of infiltration. Fatal lung disorders commonly have a DAD-like pattern, and the prognosis is poor, regardless of the drug that is used [7–9]. In contrast, almost all of our cases showed an HR-like pattern, which manifests as bilateral diffuse ground-glass opacity, responds well to steroid treatment, and has a good prognosis [7–9]. In the present study, we performed close monitoring of clinical findings, such as a continuous fever and KL-6 elevation, and CT imaging evaluation was proactively performed, which facilitated the early detection of ILD.

This study was limited by its retrospective nature and small population, which might have influenced on the incidence of ILD in our study. In addition, reporting methods of ILD may differ around the world [7]. Previous reports did not clarify whether or not PCP should be added as an ILD or pneumonitis and did not clearly distinguish between the two entities [12, 31]. If PCP had been excluded from ILD in our study, the ILD frequency in this study would have been a valid number.

In summary, we studied the clinical findings and imaging characteristics of ILD in early breast cancer patients receiving adjuvant or neoadjuvant chemotherapy. The incidence of ILD in the present study was relatively high; however, all cases were identified as mild ILDs. Dd chemotherapy increased the incidence of ILDs, partly because PCP cases were included. Proactively performing CT imaging evaluation might be useful for the early detection of ILD.

## Data Availability

The datasets used and/or analyzed during the current study are available from the corresponding author on reasonable request.

## Abbreviations

AC: Adriamycin and cyclophosphamide
AE: Adverse event
BAL: Bronchoalveolar lavage
CT: Computed tomography
CTCAE: Common Terminology Criteria for Adverse Events
DAD: Diffuse alveolar damage
dd: Dose-dense
DILD: Drug-induced interstitial lung disease
EC: Epirubicin and cyclophosphamide
eGFR: Estimated glomerular filtration rate
ER: Estrogen receptor
FEC: Fluorouracil, epirubicin, and cyclophosphamide
HER: Trastuzumab
HER2: Human epidermal growth factor receptor 2
HR: Hypersensitivity reaction
IHC: Immunochemistry
ILD: Interstitial lung disease
KL-6: Krebs von den Lungen-6
NAC: Neoadjuvant chemotherapy
OP: Organizing pneumonia
PCP: *Pneumocystis jirovecii* pneumonia
PER: Pertuzumab
PgR: Progesterone receptor
wPTX: Weekly paclitaxel
